# *Populus euphratica* Phospholipase Dδ Increases Salt Tolerance by Regulating K^+^/Na^+^ and ROS Homeostasis in Arabidopsis

**DOI:** 10.3390/ijms23094911

**Published:** 2022-04-28

**Authors:** Ying Zhang, Jun Yao, Kexin Yin, Zhe Liu, Yanli Zhang, Chen Deng, Jian Liu, Yinan Zhang, Siyuan Hou, Huilong Zhang, Dade Yu, Nan Zhao, Rui Zhao, Shaoliang Chen

**Affiliations:** 1 Key Laboratory of Forest and Flower Genetics and Breeding of Ministry of Education, College of Biological Science and Technology, Beijing Forestry University, Beijing 100083, China; zying@bjfu.edu.cn (Y.Z.); ykx0303@126.com (K.Y.); liuz6415@163.com (Z.L.); zhangyl@bjfu.edu.cn (Y.Z.); ced501@163.com (C.D.); liujian20170703@163.com (J.L.); xhzyn007@163.com (Y.Z.); housiyuan2020@163.com (S.H.); zhaonan19880921@126.com (N.Z.); ruizhao926@126.com (R.Z.); 2 Guangdong Provincial Key Laboratory of Silviculture, Protection and Utilization, Guangdong Academy of Forestry, Guangzhou 510520, China; yaojun990@126.com; 3 Forestry Institute of New Technology, Chinese Academy of Forestry, Beijing 100091, China; 4 Research Center of Saline and Alkali Land of National Forestry and Grassland Administration, Chinese Academy of Forestry, Beijing 100091, China; hlzhang2018@126.com; 5 Institute of Chinese Materia Medica, China Academy of Chinese Medical Science, Beijing 100700, China; dyu@gwdg.de

**Keywords:** antioxidant enzyme, Na^+^/H^+^ antiport, *Populus euphratica*, phospholipase Dδ, phosphatidic acid, PM H^+^-ATPase, ROS, salt stress

## Abstract

Phospholipase Dα (PLDα), which produces signaling molecules phosphatidic acid (PA), has been shown to play a critical role in plants adapting to salt environments. However, it is unclear whether phospholipase Dδ (PLDδ) can mediate the salt response in higher plants. *PePLDδ* was isolated from salt-resistant *Populus euphratica* and transferred to *Arabidopsis thaliana* to testify the salt tolerance of transgenic plants. The NaCl treatment (130 mM) reduced the root growth and whole-plant fresh weight of wild-type (WT) *A. thaliana*, vector controls (VC) and *PePLDδ*-overexpressed lines, although a less pronounced effect was observed in transgenic plants. Under salt treatment, *PePLDδ*-transgenic Arabidopsis exhibited lower electrolyte leakage, malondialdehyde content and H_2_O_2_ levels than WT and VC, resulting from the activated antioxidant enzymes and upregulated transcripts of genes encoding superoxide dismutase, ascorbic acid peroxidase and peroxidase. In addition, *PePLDδ*-overexpressed plants increased the transcription of genes encoding the plasma membrane Na^+^/H^+^ antiporter (*AtSOS1*) and H^+^-ATPase (*AtAHA2*), which enabled transgenic plants to proceed with Na^+^ extrusion and reduce K^+^ loss under salinity. The capacity to regulate reactive oxygen species (ROS) and K^+^/Na^+^ homeostasis was associated with the abundance of specific PA species in plants overexpressing *PePLDδ*. *PePLDδ*-transgenic plants retained a typically higher abundance of PA species, 34:2 (16:0–18:2), 34:3 (16:0–18:3), 36:4 (18:2–18:2), 36:5 (18:2–18:3) and 36:6 (18:3–18:3), under control and saline conditions. It is noteworthy that PA species 34:2 (16:0–18:2), 34:3 (16:0–18:3), 36:4 (18:2–18:2) and 36:5 (18:2–18:3) markedly increased in response to NaCl in transgenic plants. In conclusion, we suppose that *PePLDδ*-derived PA enhanced the salinity tolerance by regulating ROS and K^+^/Na^+^ homeostasis in Arabidopsis.

## 1. Introduction

Nowadays, soil salinity is a serious environmental problem in the world [1,2]. To adapt to such stress, plant cells initiate signaling transduction, resulting in a wide variety of molecular and physiological modulations [3,4,5]. Phospholipase D (PLD) and its lipid products, phosphatidic acid (PA), mediate salt stress signaling in higher plants [6,7,8]. Multiple PLDs—in particular, PLDα and PLDδ—are required for plants adapting to high salt environments [9]. AtPLDα1 and AtPLDδ can both hydrolyze membrane phospholipids to produce PA in response to high salt concentrations [9]. Salt stress increased transcripts of *PLDα* genes in various species, including *BnaPLDα1C1*, *BnaPLDα1A1*, *BnaPLDα1C5* and *BnaPLDα1A5* in *Brassica napus* [10], *StPLDα1*, *StPLDα4* and *StPLDα5* in *Solanum tuberosum* [11], *OsPLDα1*, *OsPLDα5* and *OsPLDα6* in rice [12] and *GmPLDα1* and *GmPLDα2* in *Glycine max* [13]. The heterologous expression of *PLDα* increased the salinity tolerance in Arabidopsis *PLDα1* knockout mutants and actively regulated the transcription of stress-responsible genes [14]. Similarly, the overexpression of *phospholipase Dα* genes from Arabidopsis (*AtPLDα*) and cucumber (*CsPLDα*) enhances the salt tolerance of *Populus tomentosa* [15] and tobacco [16,17]. In addition to *PLDα*, salt stress also increases the expression level of *PLDδ* genes in various species. For instance, both *BnaPLDδAnn* and *BnaPLDδC7* were highly induced by NaCl in *Brassica napus* [10]. *Solanum tuberosum* increased the transcripts of *StPLDδ1*, *StPLDδ2*, *StPLDδ3*, *StPLDδ4* and *StPLDδ5* under salinity [11]. *OsPLDδ1* was also found to be significantly upregulated in salt-stressed rice [12]. The expression of *GmPLDδ3* and *GmPLDδ4* in *Glycine max* transiently increased under NaCl treatment [13]. Collectively, *AtPLDδ* is involved in plant responses to dehydration stress [18]; however, whether PLDδ can mediate the salt response in higher plants is less known.

The sustained cellular K^+^/Na^+^ homeostasis is crucial for plants to tolerate salinity stress [19]. Na^+^ excretion from cells is generally carried out by Na^+^/H^+^ antiport protein SOS1 (salt overly sensitive 1) [20,21,22]. It has been shown that PLDα and PA are involved in mediating Na^+^ homeostasis under salinity. Cucumber *Phospholipase D alpha* (*CsPLDα*) overexpression enabled transgenic tobacco to retain Na^+^ balance and improved its salt tolerance [17]. Yu et al. showed that PLDα-produced phosphatidic acid (16:0–18:2) stimulates the activity of MPK6, which phosphorylates the C-terminal fragment of the Na^+^/H^+^ antiporter, SOS1. The activated SOS1 aids the plants in avoiding excess Na^+^ accumulation under salinity [23]. The plasma membrane (PM) H^+^-ATPases are fundamental for Na^+^/H^+^ exchange and salt tolerance [24,25,26,27]. NaCl increases the transcription of PM H^+^-ATPase genes in salt-resistant woody species [28,29]. The overexpression of PM H^+^-ATPase genes has been shown to improve salt tolerance in transgenic plants [26,29,30]. It is found that the over-expression of cucumber *CsPLDα* resulted in increased transcripts of a H^+^-ATPase gene, *NtNHA1*, in tobacco under short-term NaCl stress [17]. This indicates that PM H^+^-ATPases might be transcriptionally regulated by PLDα1-derived PA. Whether phospholipase Dδ affects the transcription of PM H^+^-ATPases and the salt response needs to be clarified.

In addition to Na^+^ toxicity, a high salinity leads to the excessive production of reactive oxygen species (ROS), which cause oxidative stress and destroy the membrane integrity [2,3]. Salt-resistant poplar, *P. euphratica*, can quickly activate the expression of relevant antioxidant enzyme genes and improve enzyme activity, such as superoxide dismutase (SOD), peroxidase (POD), ascorbic acid peroxidase (APX), catalase (CAT) and glutathione reductase (GR), to eliminate ROS under salt stress [31,32]. A PLDα-derived way to scavenge ROS is suggested to reduce salt stress damage. The activities of antioxidant enzymes, SOD, POD, APX and CAT, are much higher in *CsPLDα*-overexpressed plants than those of the wild-type (WT) [17]. The increased activities of SOD, POD and CAT are also observed in leaf discs of *AtPLDα*-transgenic poplar plants during salt stress [15]. Whether PLDδ upregulates the transcripts and activity of antioxidant enzymes under salinity remains unclear.

*Populus euphratica*, a typical stress-resistant tree species, is distributed in the saline alkali desert area of Northwest China. Multiple signaling molecules, including abscisic acid, ethylene, nitric oxide, hydrogen peroxide, extracellular ATP, hydrogen sulfide and calcium (Ca^2+^), are involved in mediating the salt response of *P. euphratica* [2,3,4,5]. It is unknown whether PLD and PA serve as signaling molecules in *P. euphratica* responding to salinity. To clarify this, we aimed to evaluate the role of *P. euphratica* phospholipase D in plants adapting to a salt environment. The *P. euphratica* phospholipase Dδ gene, *PePLDδ*, was cloned and heterologous expressed in the model species Arabidopsis. Transgenic lines of *A. thaliana* (*PePLDδ*-OE6 and *PePLDδ*-OE7) were used to verify whether *PePLDδ* contributes to ionic and ROS homeostasis under high salinity. The major PA species were examined in wild-type and *PePLDδ*-transgenic Arabidopsis. Our data showed that *PePLDδ* produced a higher abundance of specific PA species, 34:2 (16:0–18:2), 34:3 (16:0–18:3), 36:4 (18:2–18:2) and 36:5 (18:2–18:3), which contributed to the maintenance of ionic and ROS homeostasis in NaCl-stressed transgenic plants.

## 2. Results

### 2.1. PePLDδ Gene Cloning and Sequence Analyses

**Figure 1:**
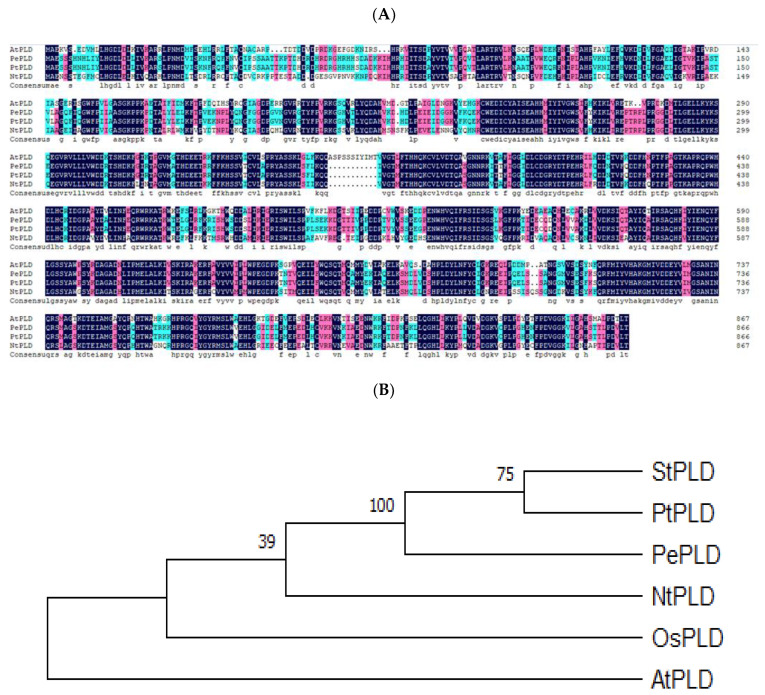
Sequence and phylogenetic analysis of *Populus euphratica* phospholipase Dδ. (**A**) Multiple sequence alignment of *Populus euphratica* PLDδ (PePLDδ) with PLDδ from different species. PePLDδ was compared with PLDδ sequences from *Populus trichocarpa* (PtPLDδ, XP_024457238.1), *Nicotiana tabacum* (NtPLDδ, XP_016456678.1), *Arabidopsis thaliana* (AtPLDδ, NP_567989.1). Black shading indicates identical amino acid residues, blue and pink shadings indicate conserved amino acids, respectively. (**B**) Phylogenetic relationships between PePLDδ and PLDδ proteins from other different species. The different species are indicated as follows: At, *Arabidopsis thaliana*; Nt, *Nicotiana tabacum*; Os, *Oryza sativa;* St, *Solanum tuberosum;* Pe, *Populus euphratica*; Pt, *Populus trichocarpa*. Accession numbers of PLDδ orthologs are shown in Supplementary Table S1.

In this study, a phospholipase Dδ gene, *PLDδ*, was cloned from *P. euphratica* leaves. The amino acid sequences were compared with PLDδ proteins from different plant species. The PePLDδ sequence displayed a high similarity to *P. tricocarpa* PLDδ (Figure 1A). The comparative phylogenetic analysis revealed that PePLDδ displayed a homology to StPLDδ and PtPLDδ, but was distinct from AtPLDδ (Figure 1B).

### 2.2. Transformation of PePLDδ Gene

**Figure 2:**
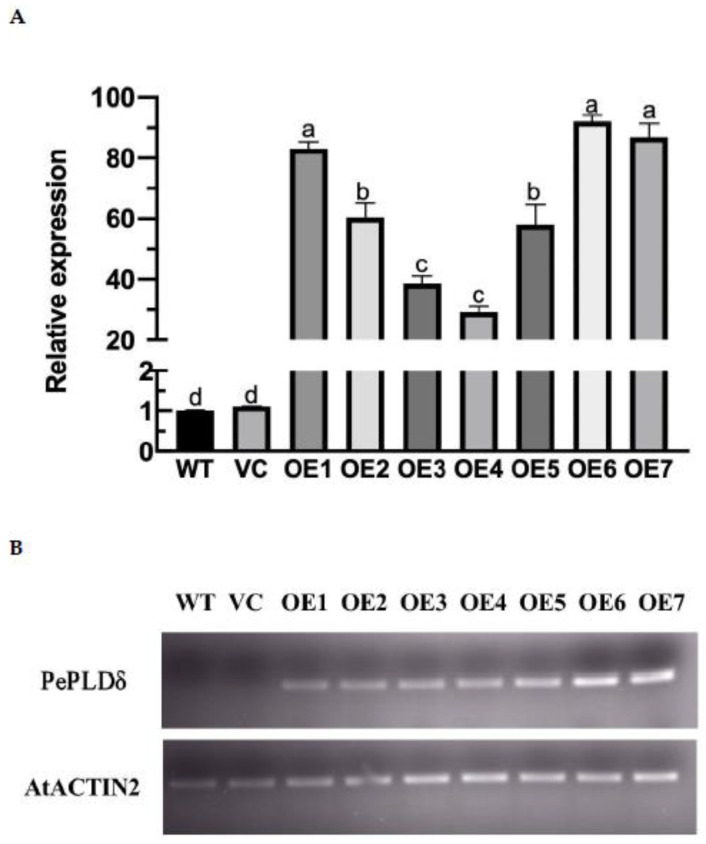
Molecular verification of transgenic Arabidopsis lines overexpressing *P. euphratica PePLDδ*. (**A**) RT-qRCR assay. (**B**) Semi-quantitative reverse transcription PCR assay. Ten-day-old seedlings of wild-type (WT) Arabidopsis, empty vector control (VC) and the transgenic lines (OE1–OE7) were sampled for total RNA extraction, semi-quantitative PCR and real-time quantitative PCR analyses. Arabidopsis *β-actin 2* (*AtACTIN2*) was used as an internal reference gene. The primers designed to target *PePLDδ* and internal control gene, *AtACTIN2*, are shown in Supplementary Table S2. In (**A**), data are presented as the mean of three independent experiments, and error bars represent SE. Columns with different letters, a, b, c and d show significant differences, with *p* < 0.05.

Seven transgenic lines, OE1, OE2, OE3, OE4, OE5, OE6 and OE7, were obtained by transferring the *PePLDδ* gene into *Arabidopsis thaliana*. The transgenic lines were identified with semi-quantitative reverse transcription PCR and real-time quantitative PCR (Figure 2). The wild-type (WT) Arabidopsis, empty vector control (VC) and two transgenic lines (OE6 and OE7) that showed the highest abundance of *PePLDδ* were used for salt treatments.

### 2.3. Salt Tolerance Tests of PePLDδ-Transgenic Arabidopsis

**Figure 3:**
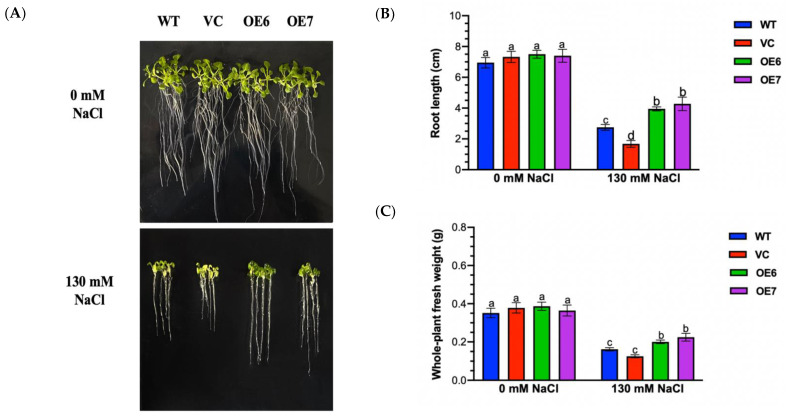
Salt tests of wild-type (WT) Arabidopsis, empty vector control (VC) and *PePLDδ*-overexpressed lines. Seeds from WT, VC and *PePLDδ*-transgenic lines (OE6 and OE7) were allowed to germinate on 1/2 MS medium and grown for seven days, and were then subjected to 0 or 130 mM NaCl treatment. Root length and whole-plant fresh weight were examined after seven days of NaCl treatment. (**A**) Representative pictures show plant performance and root lengths under control and NaCl stress. (**B**) Root length. (**C**) Whole-plant fresh weight. Data are presented as the mean of three independent experiments, and error bars represent SE. Columns with different letters, a, b, c and d in (**B**,**C**) show significant differences, with *p* < 0.05.

The change in growth can sensitively reflect the plant’s ability to adapt to a salt environment [33]. The root length and whole plant weight of all tested genotypes were compared. The root length of WT, VC and *PePLDδ*-transgenic lines, OE6 and OE7, significantly decreased upon seven days of 130 mM NaCl treatment (Figure 3A,B). The restriction of NaCl was more pronounced in WT and VC than in transgenic plants (Figure 3A,B). Moreover, the whole-plant fresh weight of salt-stressed WT and VC showed a 10–31% higher reduction than transgenic lines (Figure 3C). Our results showed that there was no significant difference between the tested lines, WT, VC and transgenic Arabidopsis, in root growth and fresh weight under no-salt conditions (Figure 3).

### 2.4. Salt-Stress-Induced Electrolyte Leakage and Membrane Peroxidation

**Figure 4:**
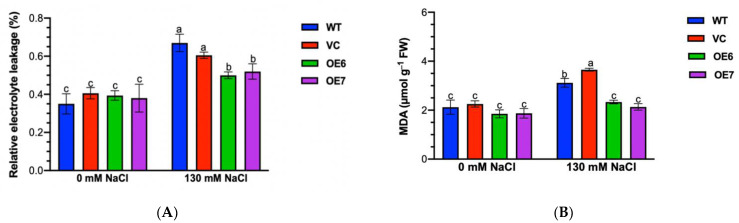
Relative electrolyte leakage and malondialdehyde (MDA) content in wild-type (WT) Arabidopsis, empty vector control (VC) and *PePLDδ*-overexpressed lines under salt stress. Seeds from WT, VC and *PePLDδ*-transgenic lines (OE6 and OE7) were allowed to germinate on 1/2 MS medium and grown for seven days, and were then subjected to 0 or 130 mM NaCl treatment. Control and salt-stressed plants were sampled to measure relative electrolyte leakage after 12 h of NaCl treatment and MDA content after 3 days of salt stress. (**A**) Relative electrolyte leakage. (**B**) MDA content. Data are presented as the mean of three independent experiments, and error bars represent SE. Columns with different letters, a, b and c show significant differences, with *p* < 0.05.

To test the salt damage effect on cell membrane integrity, the relative electrolyte leakage (EL) of control and salinized plants was examined [33]. Compared with WT and VC, *PePLDδ*-overexpressed Arabidopsis exhibited a 12–27% lower relative EL after 12 h of salt treatment (Figure 4A). Therefore, *PePLDδ* could alleviate the damage of high NaCl on the cell membrane and consequently improved the salt tolerance of transgenic Arabidopsis.

The electrolyte leakage usually results from membrane peroxidation under salt stress [33]. Here, the content of malondialdehyde (MDA), which is the end-product of membrane lipid peroxidation, was examined. The results showed that the MDA content of WT and VC significantly increased upon the salt treatment, whereas MDA remained unchanged in *PePLDδ*-overexpressed lines (Figure 4B). This indicates that *PePLDδ* reduced the oxidative damage caused by NaCl in transgenic *A. thaliana.*

### 2.5. H_2_O_2_ Content in Root Cells under Salt Stress

**Figure 5:**
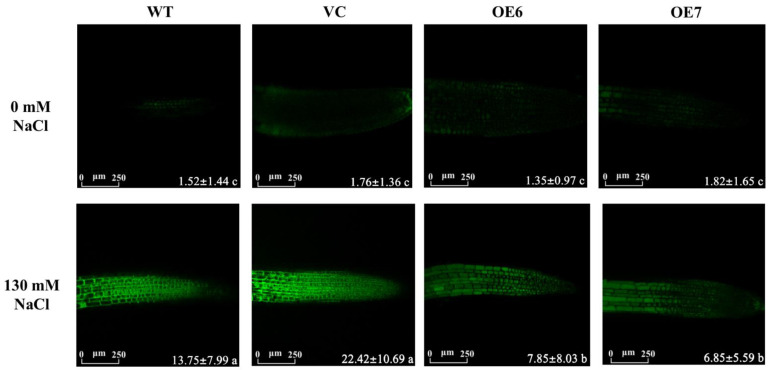
H_2_O_2_ concentrations in root cells of wild-type (WT) Arabidopsis, empty vector control (VC) and transgenic lines overexpressing *PePLDδ* under NaCl stress. Seeds from WT, VC and *PePLDδ*-overexpressed lines (OE6 and OE7) were allowed to germinate on 1/2 MS medium and grown for seven days. The seedlings were transferred to liquid medium containing 0 or 130 mM NaCl for 12 h. Then, Arabidopsis roots were incubated with 10 μM H_2_DCFDA for 15 min, followed by washing 4–5 times. Green fluorescence within cells was detected with a laser confocal microscope, and the relative H_2_O_2_ concentrations were calculated according to the fluorescence intensity. Data are presented as the mean of 6–9 individual plants, and error bars represent SE. Values with different letters, a, b, and c show significant differences, with *p* < 0.05. Scale bar = 250 μm.

Reactive oxygen species (ROS) cause membrane peroxidation and electrolyte leakage under salt stress [31,32,33]. We used the fluorescent probe, H_2_DCFDA, to detect salt-elicited H_2_O_2_, as the fluorescence intensity of intracellular DCF is positively correlated with the level of intracellular H_2_O_2_ [34]. The intensity of H_2_DCFDA showed that H_2_O_2_ levels were almost undetectable in the no-salt controls of all tested genotypes. After high salt treatment, the H_2_O_2_ level in WT and VC markedly increased in root cells, which was significantly higher than transgenic lines, OE6 and OE7 (Figure 5).

### 2.6. Activity and Transcription of Antioxidant Enzymes under Salt Stress

**Figure 6:**
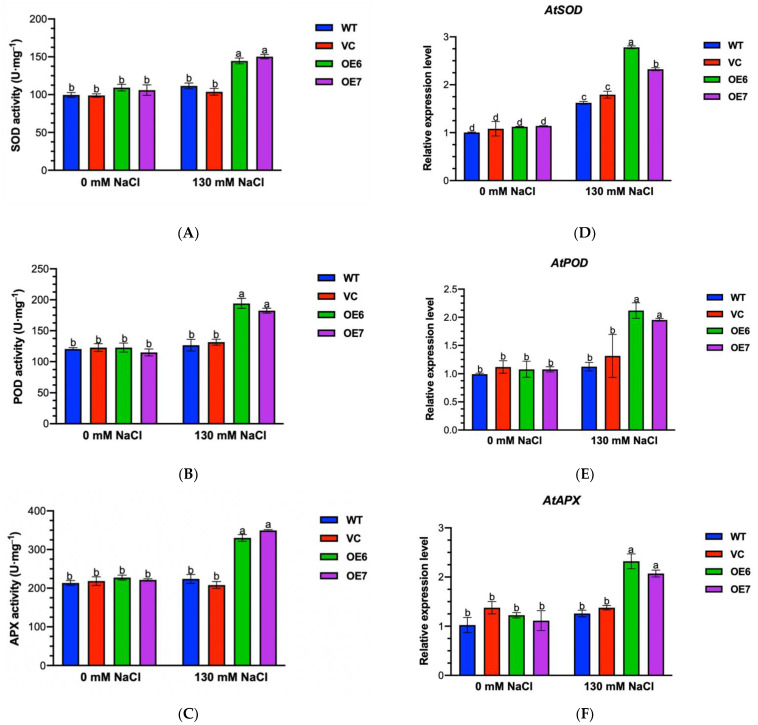
Activities of antioxidant enzymes and transcripts of encoding genes in wild-type (WT) Arabidopsis, empty vector control (VC) and transgenic lines overexpressing *PePLDδ* under NaCl stress. Seeds from WT, VC and *PePLDδ*-overexpressed lines (OE6 and OE7) were allowed to germinate on 1/2 MS medium and grown for seven days, and were then subjected to 0 or 130 mM NaCl treatment for another seven days. Total activities of ascorbic acid peroxidase (APX), peroxidase (POD), superoxide dismutase (SOD) and transcripts of encoding genes were measured after seven days of salt treatment. (**A**–**C**) Total activities of SOD, POD and APX. (**D**–**F**) Transcripts of *AtSOD*, *AtPOD* and *AtAPX*. The expression levels of *AtAPX*, *AtPOD* and *AtSOD* were detected by real-time quantitative PCR, and Arabidopsis *β-actin 2* (*AtACTIN2*) was used as the internal reference gene. Primers designed to target *AtAPX*, *AtPOD*, *AtSOD* and internal control gene, *AtACTIN2*, are listed in Supplementary Table S2. Data are presented as the mean of three repeated experiments, and error bars represent SE. Columns with different letters, a, b, c and d show significant differences, with *p* < 0.05.

The high levels of H_2_O_2_ in WT and VC mainly result from the decreased ability to scavenge ROS in salt-stressed plants [31,32,33]. To confirm whether the *PePLDδ*-transgenic lines had the capacity to maintain ROS homeostasis, the activity and transcription of antioxidant enzymes, including ascorbic acid peroxidase (APX), peroxidase (POD) and superoxide dismutase (SOD), were testified in this study. After NaCl treatment (130 mM, 7 d), the total activities of tested antioxidant enzymes, SOD, POD and APX, increased by approximately 50% in OE6 and OE7, whereas the salt stimulation was less pronounced in WT and VC (Figure 6A–C). In accordance, the expression of *AtSOD*, *AtPOD* and *AtAPX* genes showed a higher increase in *PePLDδ*-overexpressed plants compared to WT and VC, similar to the trend of enzymic activity (Figure 6D–F).

### 2.7. Na^+^ Concentration within Root Cells under Salinity Stress

**Figure 7:**
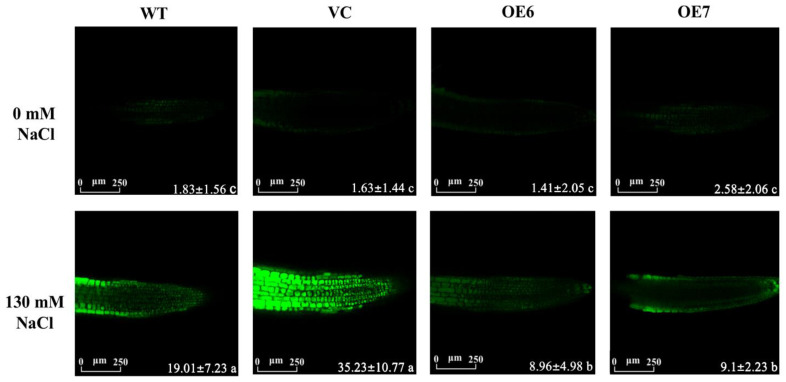
Na^+^ concentrations in root cells of wild-type (WT) Arabidopsis, empty vector control (VC) and transgenic lines overexpressing *PePLDδ* under NaCl stress. Seeds from WT, VC and *PePLDδ*-overexpressed lines (OE6 and OE7) were allowed to germinate on 1/2 MS medium and grown for seven days. The seedlings were transferred to liquid medium containing 0 or 130 mM NaCl for 12 h. Then, Arabidopsis roots were incubated with 20 μM CoroNa™ Green for 1 h, followed by washing 4–5 times. Green fluorescence within cells was detected with a laser confocal microscope, and the relative Na^+^ concentrations were calculated according to the fluorescence intensity. Data are presented as the mean of 6–9 individual plants, and error bars represent SE. Values with different letters, a, b, and c show significant differences, with *p* < 0.05. Scale bar = 250 μm.

The Na^+^ accumulation in cells results in an increase in ROS production under NaCl stress [31,32]. The content of Na^+^ in root cells was detected by a Na^+^-specific probe, CoroNa^TM^Green. Under control conditions, the fluorescence intensity of the Na^+^ probe was very low in the roots of all tested genotypes (Figure 7). After the short-term salt treatment (NaCl 130 mM, 12 h), the fluorescence intensity significantly increased in root cells (Figure 7). It is worth noting that WT and VC displayed a 2.1–3.9-fold higher CoroNa^TM^ intensity than that of transgenic plants (Figure 7), indicating the higher buildup of Na^+^ in the roots of WT and VC.

### 2.8. Na^+^ and K^+^ Fluxes under Salt Stress

**Figure 8:**
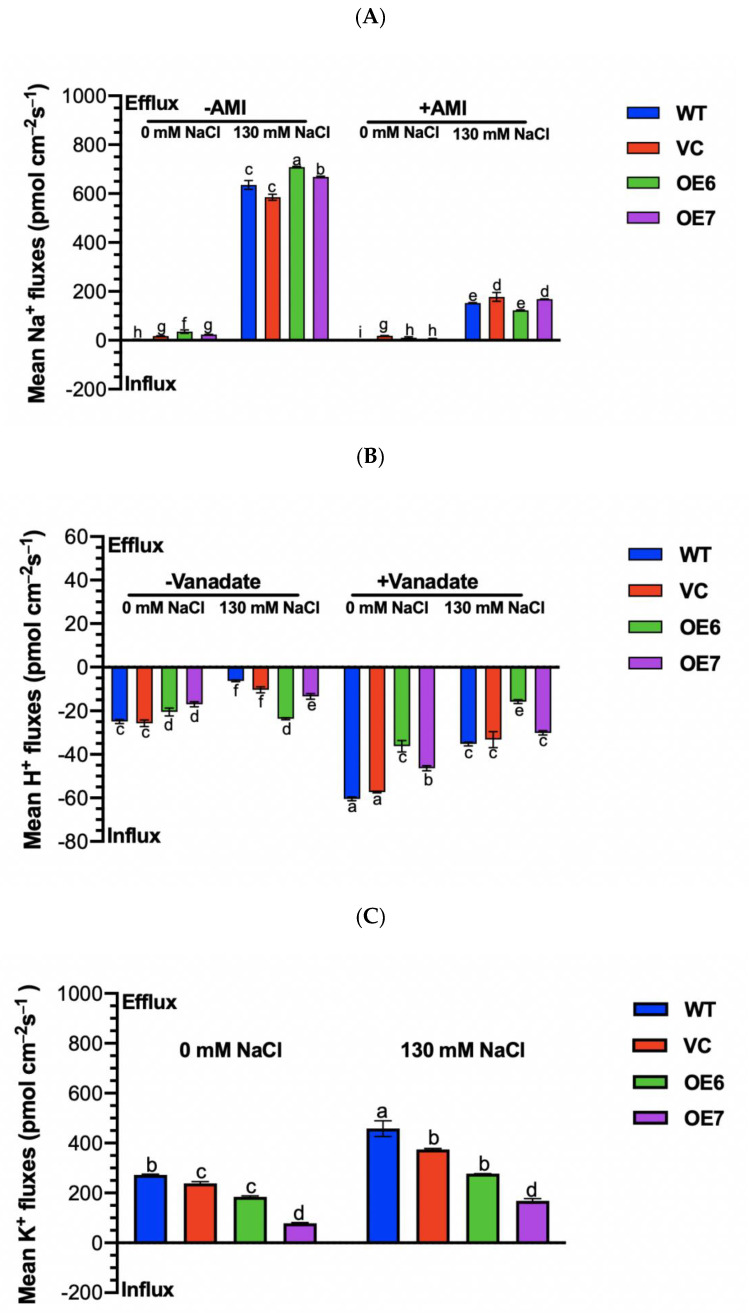
Na^+^, H^+^ and K^+^ fluxes in root tips of wild-type (WT) Arabidopsis, empty vector control (VC) and transgenic lines overexpressing *PePLDδ* under NaCl stress. Seeds from WT, VC and *PePLDδ*-overexpressed lines (OE6 and OE7) were allowed to germinate on 1/2 MS medium and grown for seven days. The seedlings were transferred to liquid medium containing 0 or 130 mM NaCl for 12 h. Then, Arabidopsis roots were incubated with amiloride (an inhibitor of Na^+^/H^+^ antiporter, 0 or 5 mM) or sodium orthovanadate (an inhibitor of plasmalemma H^+^-ATPase, 0 or 500 μM) for 30 min. Thereafter, roots were equilibrated in measuring solutions for 30 min, and Na^+^, H^+^ and K^+^ fluxes were monitored by NMT at the meristematic region (200 μm from the root tip) and continuously recorded for 10 min. (**A**) Net Na^+^ fluxes in roots treated without (−AMI) or with amiloride (+AMI). (**B**) Net H^+^ fluxes in roots treated without (−Vanadate) or with sodium orthovanadate (+Vanadate). (**C**) Net K^+^ fluxes. Data are presented as the mean of 6–9 individual plants, and error bars represent SE. Columns with different letters, a–i, show significant differences, with *p* < 0.05.

Salt-resistant species retain low Na^+^ levels by an active salt extrusion across the PM [24,25]. To confirm whether *PePLDδ*-transgenic plants could maintain Na^+^ extrusion under salinity, the Na^+^ flow in root tips was recorded with a non-invasive micro-test technique (NMT). Upon short-term exposure to NaCl (130 mM 12 h), the Na^+^ efflux increased significantly in all tested genotypes, and a higher flux rate was found in the *PePLDδ*-transgenic plants (Figure 8A). However, the Na^+^/H^+^ antiporter inhibitor, amiloride (AMI), drastically reduced the salt-elicited efflux of Na^+^ in WT, VC and *PePLDδ*-overexpressed plants (Figure 8A). The pharmacological data indicate that the Na^+^ efflux resulted from an active Na^+^/H^+^ exchange across the PM [24,25].

The H^+^ flux recordings showed that NaCl decreased the net influx of H^+^ in WT and VC, but the salt effects were less pronounced in the two transgenic lines (Figure 8B). When the specific inhibitor of PM H^+^-ATPase, vanadate, was applied, the net H^+^ influx markedly increased in Arabidopsis roots irrespective of the control and NaCl treatment (Figure 8B). Therefore, the increased net H^+^ influx was due to a decreased efflux of H^+^, which was pumped by H^+^-ATPases in the PM (Figure 8B) [26,27,29]. In comparison, the vanadate-increased H^+^ influx was lower in OE6 and OE7 than in the WT and VC in the absence and presence of NaCl (Figure 8B). This suggest that H^+^-ATPases in the PM were severely inhibited by vanadate in the root cells of WT and VC [27,29].

NaCl leads to a more pronounced K^+^ loss in salt-sensitive species than in salt-resistant species [24,25]. In this study, NaCl treatment increased K^+^ efflux in the roots of all tested lines (Figure 8C). WT and VC exhibited a 0.2–2.0-fold higher K^+^ loss than OE6 and OE7, although there is no significant difference between VC plants and the OE6 transgenic line in the mean K^+^ fluxes (Figure 8C). This shows that *PePLDδ*-transgenic Arabidopsis, and, in particular, OE7, had a greater capacity to retain K^+^ under NaCl salinity.

### 2.9. Transcription of SOS1 and AHA2 under NaCl Stress

**Figure 9:**
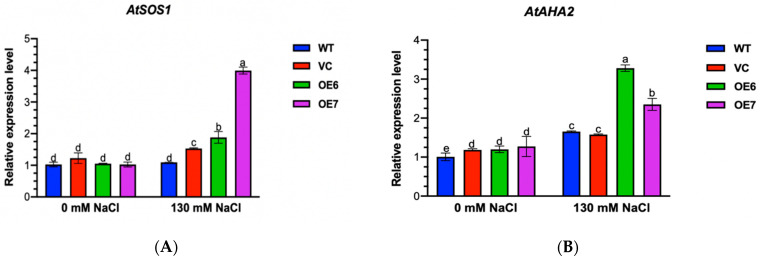
Transcription of *SOS1* (PM Na^+^/H^+^ antiporter gene) and *AHA2* (PM H^+^-ATPase gene) in wild-type (WT) Arabidopsis, empty vector control (VC) and transgenic lines overexpressing *PePLDδ* under NaCl stress. Seeds from WT, VC and *PePLDδ*-overexpressed lines (OE6 and OE7) were allowed to germinate on 1/2 MS medium and grown for seven days, and were then subjected to 0 or 130 mM NaCl treatment for 12 h. (**A**) *AtSOS1* transcription. (**B**) *AtAHA2* transcription. The expression levels of *AtSOS1* and *AtAHA2* were detected by real-time quantitative PCR, and Arabidopsis *β-actin 2* (*AtACTIN2*) was used as the internal reference gene. Primers designed to target *AtSOS1* and *AtAHA2* and internal control gene, *AtACTIN2*, are listed in Supplementary Table S2. Data are presented as the mean of three repeated experiments, and error bars represent SE. Columns with different letters, a, b, c, d, and e show significant differences, with *p* < 0.05.

It has been shown that the activated plasmalemma H^+^-ATPase and Na^+^/H^+^ antiporter result, at least in part, from the upregulated transcription of encoding genes [26,29,35,36]. Here, we examined the abundance of a typical Na^+^/H^+^ antiporter gene, *SOS1*, and a H^+^-ATPase gene, *AHA2*, in Arabidopsis plants. We observed that the transcription of *AtSOS1* and *AtAHA2* was significantly up-regulated in all tested lines under a high salt treatment (with the exception of *AtSOS1* in WT) (Figure 9A,B). It is worth noting that the salt-enhanced gene expression was more pronounced in the *PePLDδ*-overexpressed plants than in the WT and VC (Figure 9A,B). The results support the finding of a high Na^+^/H^+^ exchange in the roots of *PePLDδ*-transgenic plants (Figure 8A).

### 2.10. Phosphatidic Acid Content of PePLDδ-Transgenic Plants

**Figure 10:**
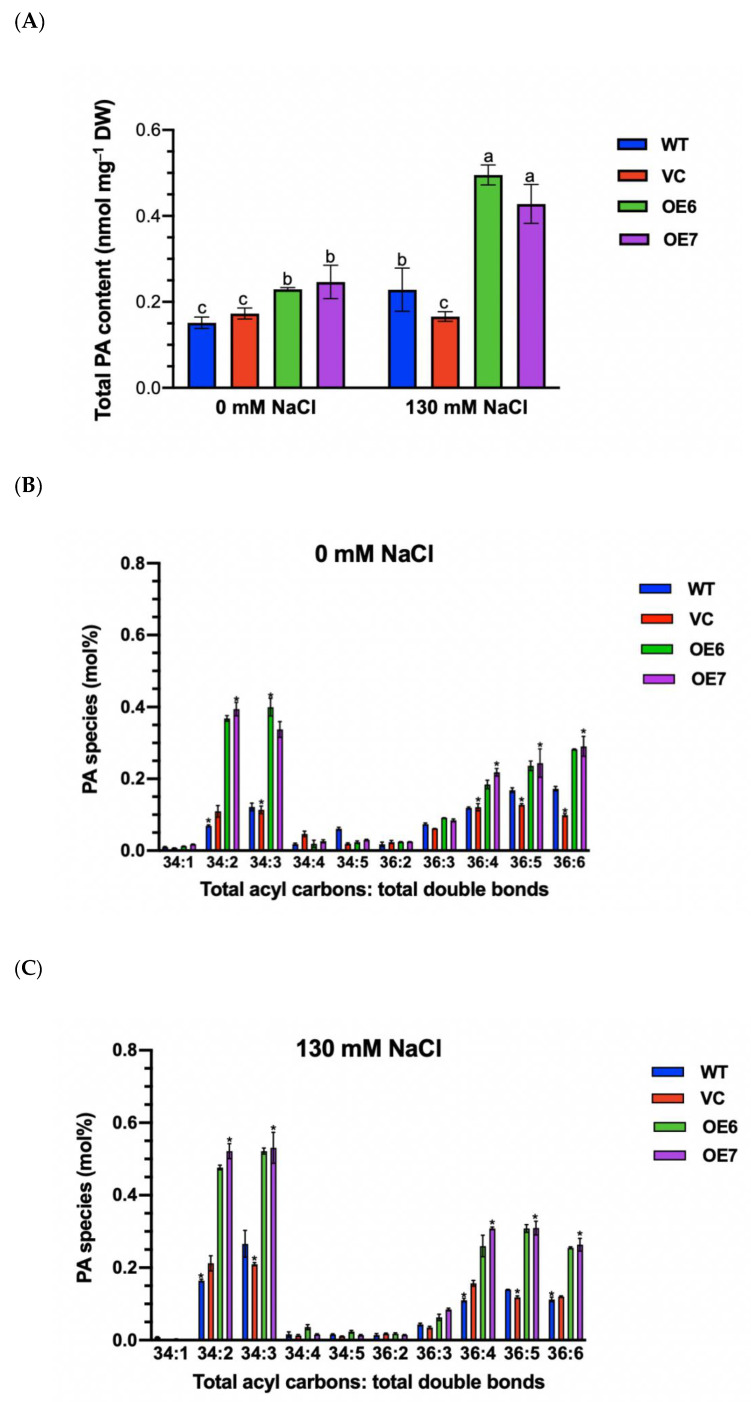
Phosphatidic acid (PA) content in wild-type (WT) Arabidopsis, empty vector control (VC) and transgenic lines overexpressing *PePLDδ* under NaCl stress. Seeds from WT, VC and *PePLDδ*-overexpressed lines (OE6 and OE7) were allowed to germinate on 1/2 MS medium and grown for seven days. Then, seedlings were transferred to liquid medium containing 0 or 130 mM NaCl for 24 h. Control and stressed seedlings were harvested, and PA species were measured using electrospray ionization–tandem mass spectrometry (ESI-MS/MS). (**A**) Total PA content. (**B**,**C**) PA species (34:1, 34:2, 34:3, 34:4, 34:4, 34:5, 36:2, 36:3, 36:4, 36:5, 36:6) in the absence (0 mM) and presence of NaCl (130 mM). Data are presented as the mean of three individual plants, and error bars represent SE. Columns with different letters, a, b and c (**A**) and asterisks (*) (**B**) show significant differences, with *p* < 0.05.

The capacity of *PePLDδ*-transgenic plants in maintaining K^+^/Na^+^ and H_2_O_2_ homeostasis might be related to phosphatidic acids (PA), the key signaling molecule in plant abiotic stress responses [6,7,8]. We measured the content of phosphatidic acids, since phospholipase D can hydrolyze membrane phospholipids to produce PA. We found that *PePLDδ*-transgenic plants retained a higher content of total PA than WT and VC (Figure 10A). It is interesting that PA significantly increased in OE6 and OE7 under salt stress, which was nearly twice that of WT and VC (Figure 10A). Moreover, the major molecular species of PA, 34:1, 34:2, 34:3, 34:4, 34:4, 34:5, 36:3, 36:4, 36:5, 36:6, were further analyzed in Arabidopsis. *PePLDδ*-overexpressed plants retained a typically higher abundance of PA species, such as 34:2 (16:0–18:2), 34:3 (16:0–18:3), 36:4 (18:2–18:2), 36:5 (18:2–18:3) and 36:6 (18:3–18:3), in the absence and presence of salt stress (Figure 10B,C). It is noteworthy that PA species 34:2 (16:0–18:2), 34:3 (16:0–18:3), 36:4 (18:2–18:2) and 36:5 (18:2–18:3) markedly increased in response to NaCl in transgenic plants (Figure 10B,C).

## 3. Discussion

### 3.1. PePLDδ Enhances Salt Tolerance in Transgenic Plants

In this study, *PePLDδ* increased the plant’s ability to tolerate salinity stress in terms of the growth response to NaCl (Figure 3). This is consistent with the reports overexpressing *PLDα* in herbaceous and woody species [14,15,16,17]. For example, the heterologous expression of the *Ammopiptanthus nanus* *PLDα* gene in Arabidopsis *PLDα1* knockout mutants enhanced the salinity tolerance of transgenic plants. Similar findings were observed in *Populus tomentosa* and tobacco overexpressing *phospholipase Dα* genes from Arabidopsis (*AtPLDα*) [15] and cucumber (*CsPLDα*) [16,17]. In this study, the *PePLDδ*-enhanced salt tolerance was associated with the increased phosphatidic acids in transgenic plants. Being the lipid products of phospholipase D, phosphatidic acids mediate salt stress signaling in higher plants [6,7,8]. Our data showed that *PePLDδ*-overexpressed plants retained a typically higher abundance of PA species, 34:2 (16:0–18:2), 34:3 (16:0–18:3), 36:4 (18:2–18:2), 36:5 (18:2–18:3) and 36:6 (18:3–18:3), under control and stress conditions (Figure 10). Furthermore, PA species 34:2 (16:0–18:2), 34:3 (16:0–18:3), 36:4 (18:2–18:2) and 36:5 (18:2–18:3) markedly increased in response to NaCl in transgenic plants (Figure 10). This agrees with the result of Yu et al. (2010), who found that PA species 34:2, 34:3, 34:6, 36:3 and 36:6 increased upon NaCl exposure [23]. Among these species, the specific PA 34:2 (a 16:0–18:2 PA) was suggested to play a crucial role in mediating the salt stress signal transduction [23]. Accordingly, the increased PA species in *PePLDδ*-transgenic plants could mediate the plant salt stress response. Therefore, *PePLDδ* overexpression positively regulates the plant’s tolerance to NaCl salinity. Our data showed that *Populus euphratica* phospholipase Dδ increases salt tolerance by regulating K^+^/Na^+^ and ROS homeostasis in Arabidopsis.

### 3.2. PePLDδ Mediates ROS Homeostasis under Salt Stress

Salinity increased the production of reactive oxygen species, e.g., H_2_O_2_ (Figure 5), which destroyed the integrity of the cell membrane, leading to solute leakage (Figure 4, [31,32,33]). *PePLDδ*-transgenic plants retained low levels of H_2_O_2_, MDA and EL under salt stress (Figure 4 and Figure 5). Similarly, the MDA content and ROS (O_2_^−^· and H_2_O_2_) production are much lower in tobacco overexpressing cucumber *CsPLDα* under NaCl stress compared to WT plants [17]. In addition, the *AtPLDα*-transgenic *P. tomentosa* plants displayed a greater capacity in scavenging ROS than the wild-type [15]. The overexpression of *PePLDδ* upregulated activities of antioxidant enzymes, such as APX, SOD and POD (Figure 6), which scavenged the salt-elicited excessive ROS, thus reducing the ROS-induced membrane oxidation (Figure 4 and Figure 5). Therefore, PePLDδ constitutes a signaling cascade controlling ROS and improving the salinity tolerance in transgenic plants. Similarly, in Arabidopsis, PLD and PA decrease the cell death induced by H_2_O_2_ [37]. We suppose that the increase in PA in *PePLDδ*-overexpressed plants might initiate the salt signaling cascade in retaining the ROS homeostasis. It is suggested that PLD and PA mediate the generation of superoxide in Arabidopsis [38,39]. Moreover, Arabidopsis PLDδ was shown to transduce H_2_O_2_ signaling by interacting with glyceraldehyde-3-phosphate dehydrogenases under stress conditions [40]. The PePLDδ- and PA-stimulated ROS might activate the antioxidant enzymes, i.e., SOD, APX and POD, in salt-stressed plants overexpressing *PePLDδ*, since reactive oxygen species have been implicated as second messengers to induce antioxidant defenses [41,42]. We have previously shown that the activated SOD, APX and GR in salt-resistant *P. euphratica* was associated the rapid increase in ROS (O_2_^−^· and H_2_O_2_) after the onset of salt treatment [31,32]. In accordance, *CsPLDα*-overexpressed tobacco plants displayed much higher activities of SOD, POD, CAT and APX than those of the wild-type [17]. The activated SOD, CAT and POD were also observed in leaf discs of *AtPLD*-overexpressed poplars during NaCl treatment [15]. Under high salinity, the up-regulated expression of *SOD*, *APX* and *POD* also contributed to the enzyme activity in plants overexpressing *PePLDδ* (Figure 6). The interaction between *PePLDδ*, PA and these antioxidant enzymes needs to be further investigated.

### 3.3. PePLDδ Mediates K^+^/Na^+^ Homeostasis under Salt Stress

*PePLDδ*-overexpressed plants retained Na^+^ homoeostasis under salt stress (Figure 7) as a result of the greater Na^+^ extrusion from the root cells (Figure 8). The increased gene expression of *AtSOS1* and *AtAHA2* in transgenic lines suggests that the Na^+^ efflux resulted from an active Na^+^/H^+^ exchange promoted by the PM H^+^-ATPase (Figure 9) [35,36]. It is suggested that the NaCl-increased specific PA species, i.e., 16:0–18:2, were able to interact with mitogen-activated protein kinase 6 (MPK6), which directly phosphorylates the downstream Na^+^/H^+^ exchanger SOS1 under a high salt condition [23]. Accordingly, *PePLDδ*-derived PA species, 34:2 (16:0–18:2), in transgenic plants could also activate *AtSOS1* through a MAPK signaling pathway, thus promoting the Na^+^ extrusion via a Na^+^/H^+^ exchanger across the plasma membrane. Moreover, it has been proposed that the PLD-produced PA activated the H^+^-ATPase and Na^+^/H^+^ exchanger in the vacuolar membrane to increase the salinity tolerance [43]. We suggest that *PePLDδ*, similar to *PLDα*, enhanced the PM H^+^-ATPase and Na^+^/H^+^ antiporter to extrude Na^+^ from salt-stressed roots. However, how *PePLDδ* and PA activate the Na^+^/H^+^ antiport system in the PM needs to be further clarified.

The lower K^+^ loss in *PePLDδ*-overexpressed plants, particularly OE7, was presumably related to the PM H^+^-ATPase (Figure 8 and Figure 9). The upregulation of *AtAHA2* resulted in an increased activity of H^+^-pumps, as PM H^+^-ATPases are transcriptionally regulated in transgenic lines [26,29], in addition to post-translational modulation [27]. The activated H^+^-pumps not only promoted Na^+^/H^+^ exchange but also hyperpolarized the membrane potential, thus reducing the K^+^ loss through depolarization-activated channels, e.g., outward rectifying potassium channels and non-selective cation channels [24,25]. Consequently, *PePLDδ*-overexpressed plants increased their ability to retain K^+^/Na^+^ homeostasis under NaCl stress. Similarly, Ji et al. suggest that the proton-pumps activated by cucumber *CsPLDα* and *CsPLDα*-produced PA that enabled transgenic tobacco plants to maintain K^+^/Na^+^ homeostasis under salinity stress [17]. We noticed that the transgenic line OE7 exhibited a greater capacity than OE6 to retain K^+^ under salt stress. This might be related to the higher level of specific PA 34:2 in *PePLDδ*-OE7 (Figure 10), which serves as a critical molecule that mediates salt stress signaling [23]. Nevertheless, long-term experiments are needed to evaluate the transgenic lines’ capability to tolerate long-term salt stress conditions, produce flowers and set fruits.

## 4. Materials and Methods

### 4.1. Culture of Populus euphratica and Arabidopsis thaliana

*P. euphratica* seedlings (1-year-old) from Xinjiang Uygur Autonomous Region, China were raised at a greenhouse of Beijing Forestry University (BFU). The plants were well-irrigated and fertilized during three months of culture [24,44]. Upper mature leaves were sampled for total RNA isolation and *PePLDδ* gene cloning.

*Arabidopsis thaliana* were seeded in 1/2 MS agar medium and cultured in climate chamber after 3 d of low-temperature stratification treatment. The temperature was 22 ± 1 °C and humidity was maintained at 50–60%. Photosynthetically active radiation was 150 µmol m^−2^ s^−1^ during a long-day photoperiod (16 h). After 10 days of culture in plates, the *A. thaliana* seedlings with 4 cotyledons were planted in 200 mL pots containing nursery soil and vermiculite in a ratio of 1:1, and placed in the culture room at BFU.

### 4.2. Cloning of PePLDδ Gene

Total RNA for first-strand cDNA synthesis was extracted from *P. euphratica* leaves using the TRIzol reagent (Invitrogen, Carlsbad, CA, USA) in accordance with the manufacturer’s instructions. RNA (1 μg) was then reverse transcribed using Oligo dT adaptor primers (Promega, Madison, WI, USA). The gene sequence of *P. euphratica* phospholipase Dδ (Pe; reference sequence number XM_011023928.1 on NCBI) was used to design primers. The primer sequences (5′-to-3′) were as follows: forward, ATG GCT GAG CTC CAG TCA AC; reverse, TTA TGT TAA CAT CGG GAA G. The PCR processes for full-length gene cloning were followed as previously described [45,46]. The PCR products were purified and cloned into the pMD18-T vector (Takara, Kusatsu, Japan), and then the recombinant plasmid was transformed into *Escherichia coli* Top10 competent cells (Invitrogen, Carlsbad, CA, USA). *E. coli* bacterial cells were grown on LB sterile agar medium, and the ampicillin-resistant single colonies were cultured in liquid medium to obtain the full-length of *PePLDδ*.

### 4.3. Sequence and Phylogenetic Analyses

We performed PLDδ protein multiple-sequence alignments using ClustalW (http://www.genome.jp/tools/clustalw/, accessed on 18 August 2020, EMBL-EBI, Hinxton, Cambridgeshire, UK). The phylogenetic tree was determined with MEGA 5.2 software (http://www.megasoftware.net/index.php, accessed on 18 August 2020, Center for Evolutionary Medicine and Informatics, Tempe, AZ, USA). Accession numbers of PLDδ orthologs used in multiple-sequence alignment and phylogenetic analysis are shown in Supplementary Table S1.

### 4.4. Construction and Screening of PePLDδ-Transgenic Lines

Transformation of *Arabidopsis thaliana* was performed by flower dipping method. The full-length *PePLDδ* gene was inserted into the expression vector pMDC85, containing cauliflower mosaic virus 35S (*CaMV 35S*) promoter to obtain recombinant plasmids pMDC85-*PePLDδ*. The cloned plasmid with the target gene was transformed into *Agrobacterium tumefaciens.* The flower buds of four-week-old soil-cultivated *Arabidopsis thaliana* were immersed in the bacterial solution for 5–10 s, then cultured at 22 ± 1 °C for 16–24 h in darkness. Thereafter, the infected plants were transferred to Arabidopsis culture room and well-watered until mature seeds were collected. The seeds were dried for 1 week and used for antibiotic screening. The antibiotic-resistant plants were grown in nutrient soil to obtain homozygous T3 generation seeds. The transgene expression levels of seven T3 homozygous lines, OE1, OE2, OE3, OE4, OE5, OE6 and OE7, were quantified with semi-quantitative reverse transcription PCR and RT-qPCR.

### 4.5. Phenotype Tests of Transgenic Plants

Phenotypic screening of *PePLDδ*-transgenic *Arabidopsis thaliana* under NaCl salinity was performed in plates with 1/2 MS medium. Seeds of wild-type (WT) Arabidopsis, empty vector control (VC) and transgenic lines overexpressing *PePLDδ*, OE6 and OE7 were sterilized with 1% sodium hypochlorite for 10 min, followed by washing 5–6 times, and sown in 1/2 MS medium. After vernalization treatment for 3–5 days, the *A. thaliana* seedlings were transferred to 1/2 MS medium supplemented with 0 or 130 mM NaCl. The petri dishes were vertically placed, and photos were taken for root length measurement after seven days of salinity treatment. The root length was measured using image processing software ImageJ pro6 (http://rsb.info.nih.gov/ij/, accessed on 8 October 2021). The fresh weight of *A. thaliana* seedlings was obtained immediately after the plants were harvested. Salt tolerance tests for transgenic lines were repeated three times.

### 4.6. Electrolyte Leakage and MDA Measurement

Mature leaves were sampled from seedlings of WT Arabidopsis, VC and transgenic lines overexpressing *PePLDδ* after treatment without or with 130 mM NaCl. The initial conductivity and final conductivity of leaf samples were examined to determine the relative electrolyte leakage (EL) as previously described [33,45].

To measure MDA content, 1 g (fresh weight) of leaves was sampled from control and NaCl-treated plants of WT, VC and *PePLDδ*-overexpressed lines. Samples were immersed in 10 mL 10% trichloroacetic acid (TCA) for full grinding, then centrifuged at 4000 rpm for 10 min. Two milliliters of the supernatant was mixed with the same volume of 0.6% 2-thiobarbituric acid (TBA) solution and boiled for 20 min. For blank controls, 2 mL distilled water was mixed with TBA solution. After cooling to room temperature, the absorbance was measure at 450, 532 and 600 nm, respectively. The MDA concentration (µmol L^−1^) was calculated as: 6.45 × (D532-D600) − 0.56 × D450 [33,45].

### 4.7. Real-Time Quantitative PCR

Seedlings of WT Arabidopsis, VC and *PePLDδ*-overexpressed lines treated without or with 130 mM NaCl were harvested for RT-qPCR analysis. The Trizol reagent (Invitrogen, Carlsbad, CA, USA) and EASYspin Plus Plant RNA Kit (Aidlab Biotech, Beijing, China) were used to isolate total RNA from Arabidopsis leaves. Based on the manufacturer’s recommended protocol, RNA (1 μg) was used for reverse transcription with Moloney murine leukemia virus (M-MLV) reverse transcriptase and an oligo (dT) primer (Promega, Madison, WI, USA). The resulting cDNA products were used as templates for RT-qPCR. We used Arabidopsis *β-actin 2* (*AtACTIN2*) as the internal reference gene. Forward and reverse primers designed to target *AtSOS1*, *AtAHA2*, *AtSOD*, *AtPOD*, *AtAPX* and internal control gene, *AtACTIN2*, are listed in Supplementary Table S2. The composition of reaction mixture and running conditions for RT-qPCR have been described elsewhere [33,45,46]. Each sample was repeated at least three times. The relative target gene expression level was normalized to the reference gene *AtACTIN2* using the cycle threshold (Ct) values [47].

### 4.8. Na^+^, K^+^ and H^+^ Fluxes in Roots

Na^+^, K^+^ and H^+^ fluxes in Arabidopsis roots were recorded with non-invasive micro-test technique (NMT-YG-100, Younger USA, LLC, Amherst, MA 01002, USA) equipped with selective microelectrodes for target ions [27,29,33]. Seven-day-old Arabidopsis seedlings of WT, VC and *PePLDδ*-overexpressed lines (OE6 and OE7) were exposed to 0 or 130 mM NaCl for 12 h. Then, Arabidopsis roots were incubated with amiloride (an inhibitor of Na^+^/H^+^ antiporter, 0 or 5 mM) or sodium orthovanadate (an inhibitor of plasmalemma H^+^-ATPase, 0 or 500 μM) for 30 min. Thereafter, roots were sampled and equilibrated for 30 min in the basic solution containing 0.1 mM NaCl, 0.1 mM CaCl_2_, 0.1 mM MgCl_2_, 0.5 mM KCl and 2.5% sucrose (pH 5.8). Na^+^, H^+^ and K^+^ fluxes were monitored by NMT microelectrodes at the meristematic region (200 μm from the root tip) and continuously recorded for 10 min.

### 4.9. Na^+^ and H_2_O_2_ Contents in Root Cells

Na^+^ specific probe, CoroNa^TM^Green AM (Invitrogen, Carlsbad, CA, USA), was used to measure Na^+^ concentrations within Arabidopsis root cells. Seven-day-old seedlings of WT, VC and *PePLDδ*-overexpressed lines (OE6 and OE7) grown on 1/2 MS medium were exposed to 0 or 130 mM NaCl for 12 h. The control and NaCl-stressed roots were incubated in CoroNa^TM^Green (20 µM) in 5 mM MES/KCl loading buffer (pH 5.7) [27,33]. After 2 h incubation in darkness, roots were rinsed with 1/2 MS solution 4–5 times. For H_2_O_2_ assay, Arabidopsis roots were incubated in 10 µM H_2_DCFDA (Molecular Probe, Eugene, OR, USA) for 15 min and washed 4–5 times before confocal analysis [25,33,35]. Leica SP8 confocal microscope was used to detect the fluorescence intensity, with excitation wavelength 488 nm and emission wavelength 510–530 nm [25,33,35]. The relative fluorescence intensity of Na^+^ and H_2_O_2_ was analyzed quantitatively in Image Pro Plus 6.0 (Media cybernetics, silver spring, Rockville, MD, USA).

### 4.10. Determination of Antioxidant Enzyme Activity

The seeds from WT, VC and *PePLDδ*-overexpressed lines (OE6 and OE7) were germinated and cultured on 1/2 MS medium for seven days. These seedlings were then treated with 0 or 130 mM NaCl for another seven days. Control and salt-stressed seedlings were harvested and ground in liquid-nitrogen-precooled mortars. The samples (0.1 g fresh weight) were mixed with 1 mL precooled extraction buffer containing 1mM EDTA, 1% PVP, 1mM ASA and 50 mM potassium phosphate buffer (pH 7.0). Through centrifugation (12,000 g) at 4 °C for 10 min, the supernatant solution was obtained to determine enzyme activities of superoxide dismutase (SOD), peroxidase (POD) and ascorbic acid peroxidase (APX). Antioxidant enzyme activity assay kits, such as A001-3-2 (total SOD determination kit), A084-3-1 (POD assay kit) and A123-1-1 (APX test box) (Nanjing Jiancheng Bioengineering Institute, Nanjing, China), were used to detect enzymatic activity according to the manufacturer instructions. The total protein in crude enzyme extract was assayed with A045-2-2 (total protein determination kit) (Nanjing Jiancheng Bioengineering Institute, Nanjing, China).

### 4.11. Phosphatidic Acid Species Analysis

Seeds from WT, VC and *PePLDδ*-overexpressed lines (OE6 and OE7) were allowed to germinate on 1/2 MS medium and grown for seven days. Then, seedlings were transferred to liquid medium containing 0 or 130 mM NaCl for 24 h. Control and salinized Arabidopsis plants were harvested, frozen in liquid nitrogen and used to measure phosphatidic acids. The lipid was extracted and phosphatidic acids were analyzed and quantified by means of electrospray ionization–tandem mass spectrometry (ESI-MS/MS) as previously described [48].

### 4.12. Data Analysis

Na^+^, K^+^ and H^+^ fluxes were calculated using the program JCal V3.2.1, a free MS Excel spreadsheet developed by Yue Xu (http://www.xuyue.net/, accessed on 5 May 2021). All of the experimental data were subjected to SPSS version 19.0 (IBM Corporation, Armonk, NY, USA) for tests of normality and homogeneity of variances. One-way ANOVA was applied to compare the means between different treatments. Post hoc test was performed using S-N-K method. *p* < 0.05 was considered significant unless otherwise noted.

## 5. Conclusions

We propose that *PePLDδ* overexpression positively regulates plant tolerance to NaCl salinity. The *PePLDδ*-enhanced salt tolerance was associated with an increased PA in transgenic plants. *PePLDδ*-overexpressed plants retained a typically higher abundance of PA species, 34:2 (16:0–18:2), 34:3 (16:0–18:3), 36:4 (18:2–18:2), 36:5 (18:2–18:3) and 36:6 (18:3–18:3), under salt and no-salt control conditions (Figure 10). It is noteworthy that PA species 34:2 (16:0–18:2), 34:3 (16:0–18:3), 36:4 (18:2–18:2) and 36:5 (18:2–18:3) markedly increased in response to NaCl in transgenic plants. The phospholipase Dδ- and PA-mediated generation of ROS might increase the activities and transcription of SOD, APX and POD in *PePLDδ*-transgenic plants, since reactive oxygen species act as intracellular second messengers in mediating antioxidant defenses. The PePLDδ-produced PA contributed to restricting excessive Na^+^ accumulation by enhancing the H^+^-ATPase-promoted Na^+^/H^+^ exchange in the PM. The lower K^+^ loss in *PePLDδ*-overexpressed plants was presumably related to the PM H^+^-ATPase. Being signaling molecules, *PePLDδ*-derived PA increases the salt tolerance by regulating K^+^/Na^+^ and ROS homeostasis in Arabidopsis. Future experiments are devoted to investigating the nature of these results from genetic, biochemical and physiological points of view. 

## Data Availability

The data presented in this study are available in the article and Supplementary Materials.

## Supplementary Materials

The following are available online at https://www.mdpi.com/article/10.3390/ijms23094911/s1.
