# Supplementary material for: Populus euphratica Phospholipase Dδ Increases Salt Tolerance by Regulating K+/Na+ and ROS Homeostasis in Arabidopsis
Source: Int J Mol Sci. 2022 Apr 28;23(9):4911. doi: 10.3390/ijms23094911 (PMC9105705; doi:10.3390/ijms23094911)
Supplement: Supplementary file 1 [file ijms-23-04911-s001.zip › ijms-1676663-supplementary.pdf]

Table S1. Accession numbers of PLDδ orthologs used in multiple sequence alignment and phylogenetic analysis.

Sequence name	Accession number
AtPLDδ	NP_567989.1
PePLDδ	XM_011023928.1
NtPLDδ	XP_016456678.1
PtPLDδ	XP_024457238.1
StPLDδ	XP_006340788.1
OsPLDδ	XP_015647988.1

Table S2. Primers used for quantitative real-time PCR

Gene	Forward primer (5′–3′)	Reverse primer (5′–3′)
AtSOD	AGGAAACATCACTGTTGGAGAT	GAGTTTGGTCCAGTAGAGGGAA
AtPOD	CGTGCCCTTCATATTGTTGG	GACGCCATCAACAACGAGTC
AtAPX	AATATGCTGACGATGAGGATGC	CAAGAATCAAGGAGGTAGGAGATG
AtSOS1	GTGAAGCAATCAAGCGGAAA	TGCGAAGAAGGCGTAGAACA
AtAHA2	TGACTGATCTTCGATCCTCTCA	GAGAATGTGCATGTGCCAAA
PePLDδ	TGATGGGCCAGCTGCATATGAT	AGGTGGAACTATTGTGGTTCCATCC
AtACTIN2	GGTAACATTGTGCTCAGTGGTGG	AACGACCTTAATCTTCATGCTGC
